# Mechanical Characterization of Sustainable Mortars with Recycled Aggregates from Construction and Demolition Wastes: An Experimental Investigation

**DOI:** 10.3390/ma17225409

**Published:** 2024-11-05

**Authors:** Luca Soldati, Michele Bacciocchi, Angelo Marcello Tarantino

**Affiliations:** 1 Department of Economics, Science, Engineering and Design, University of the Republic of San Marino, 47891 Dogana, San Marino; luca.soldati@unimore.it; 2 Engineering Department “Enzo Ferrari”, Università degli Studi di Modena e Reggio Emilia, 41125 Modena, Italy; angelomarcello.tarantino@unimore.it

**Keywords:** sustainability, construction materials, recycled aggregates, mortar, experiments

## Abstract

The use of recycled aggregates in the production of concrete and mortar represents a sustainable way to reintroduce these constituents—which are typically treated as waste and disposed of—in the production chain, providing new value to potentially polluting materials. The effect of recycled aggregates has been widely studied in the production of concrete due to the directions of National Standards in Italy; however, their role in the manufacturing of mortar must be investigated further due to the high variability that can be observed in the literature. In particular, the aim of this paper is the mechanical characterization of sustainable mortars defined by different mix designs and different binders, in which the aggregates are gradually replaced by a recycled sand obtained from the grinding of construction and demolition wastes, which could include old concrete, clay bricks, and minimal amounts of other kinds of residual materials. This investigation is carried out through experimentation, taking into account four different mortar compositions defined by an increasing percentage of recycled constituents. Virgin aggregates are also studied for the sake of comparison. The results, accomplished through a three-point bending test and an unconfined compression test, show that it is still possible to maintain acceptable mechanical properties by using these wastes as aggregates in spite of a decrease in the analyzed values. In general, the mean reductions with respect to the use of natural aggregates are about 30–40% and 35–55%, respectively, for compressive and flexural strengths. It should be highlighted that some experimental sets provide a maximum reduction of 70–80%, but the results are still within the limitations of the standards. This aspect can be considered to be a good compromise since the production of this sustainable construction material can represent a solution that is able to reduce the extreme exploitation of natural resources, the pressure on landfills, and the consumption of energy, which are related to the construction industry.

## 1. Introduction

Currently, it has been estimated that the building sector accounts for up to 33% of global emissions, approximately 40% of material consumption, and 40% of waste generation [1,2]. Urban systems often manifest linear material flows and inefficient resource utilization, contrary to the principles of a circular economy. Concrete stands out as the predominant construction material, ranking as the second-most utilized substance on Earth, following water; its use is expected to increase significantly in the coming years. Furthermore, the International Energy Agency (IEA) asserts that the cement sector ranks third in energy consumption and second in carbon dioxide emissions among industrial sectors. These emissions have surged to around 8%, nearly tripling the 2020 levels of approximately 3%, primarily attributable to the expanding construction sector [3].

The increasing demand for concrete comes with a rise in the annual extraction of natural resources, such as natural stones and aggregates, comprising 60–75% of concrete total volume. The yearly extraction volume of sand and gravel ranges from 32 to 50 million tons [4], predominantly sourced from aquatic environments like rivers. As highlighted in the critical paper by Rentier and Cammeraat [5], this tendency can only increase in the next decades, causing effects on the physical and biological environments. Other environmental impacts related to the process of natural sand exploitation are discussed in the paper by Dan Gavriletea [6]. For this reason, the research community has pushed into the use of recycled aggregates in the production of more sustainable construction materials, opening a wide range of possibilities in the reuse of these constituents due to the environmental benefits that can be attained [7,8]. Extensive research has been conducted in recent years on the use of recycled aggregates in the production of concrete, and an exhaustive review of the most important works in this field could be found in a paper by Wang et al. [9], where a qualitative discussion of recycled aggregates is presented. As far as sustainable concrete is concerned, recent studies about shot-earth technology should be mentioned as a valid alternative to produce cementitious materials by embedding earthen constituents [10,11]. Shot-earth is a sustainable method for producing a material that can be employed in both structural and non-structural applications. By adding a stabilizer to the mixture, it is possible to achieve properties similar to those of low-strength concrete. In this context, several investigations are carried out to assess its mechanical features [12,13,14] and physical properties [15,16,17].

It should be emphasized that the research concerning the production of sustainable mortar began more recently. In fact, as stated by Restuccia et al. [18], the fine fraction of recycled aggregates has recently gained the attention of the scientific community, since the resulting sand in the recycling process has represented an increasingly remarkable portion of construction and demolition wastes. In the following paragraphs, a brief literature review concerning the main achievements in this topic is presented for the sake of completeness.

Srivastava and Singh [19] provided a comprehensive overview of studies that incorporate different types of alternative sand in the production of mortar, such as coal bottom ash, crushed rock sand, copper sand, foundry sand, and recycled fine aggregate, highlighting their physical, chemical, and mechanical properties. This discussion can be taken as a starting point to further expand the research is this direction. For example, as specified by Torkittikul et al. [20], coal bottom ash (waste from thermal power plant) can be investigated as a potential recycled aggregate as well. In particular, they found that the mechanical properties of the mortar, such as compressive strength, keep an adequate value with a replacement ratio of up to 50%; beyond this ratio, the mechanical properties tend to decrease. Wastes generated from foundry could be also used in the production of new sustainable mortar, as specified in the work by Siddique and Singh [21].

In terms of mechanical properties of hardened mortars, if fine aggregates coming from construction and demolition wastes are employed, various tendencies have been observed depending on the percentage of recycled sand introduced in the mixture. Opposing results have been also obtained. For instance, higher replacement levels of natural sand have not always determined lower mechanical features, as expected. The main causes of these variations are strictly related to the composition of the provided recycled aggregates. In fact, as stated in the paper by Poon et al. [22], the heterogeneity of construction and demolition wastes entails a remarkable variability in the mechanical properties since the recycled constituents are characterized by different features. This fact represents one of the major challenge that could limit their use and complicate the prediction of the mechanical performance of mortars with recycled aggregates. To this aim, a huge statistical analysis and a performance-based classification of recycled aggregates have been presented by Silva et al. [23]. In this framework, Fernandez Ledesma et al. [24] have analyzed the maximum feasible replacement ratio of natural sand, suggesting a value of 50% for this aim. Similar results have been presented by Ma et al. [25]. On the other hand, in a paper by Neno et al. [26] a replacement value equal to 20% is specified. A similar conclusion can be observed in the analysis by Dapena et al. [27]. This percentage must be further reduced according to the results presented by Braga et al. [28]. Nasr et al. [29], instead, noticed a significant improvement in the mechanical properties if the natural sand is fully replaced by recycled aggregates. These few references clearly prove that the mechanical properties of mortars with recycled aggregates are not always coherent and consistent with other available results not only due to the heterogeneous nature of wastes but also due to the origin of wastes.

In general, a greater demand of water has been observed when the percentage of recycled sand is higher, especially if the mixes include particles from concrete and brick debris. In this context, the paper by Thang et al. [30] should be taken as a reference for the study of the effects of the moisture conditions on mortar based on recycled sand. A common tendency has been observed, which is the typical reduction in workability associated with the presence of these aggregates. Although the mechanical properties exhibit a remarkable variability and are strictly related to the type and origin of recycled aggregates, the workability and water demand are coherent across most of the studies available in the literature. It should be specified that the reduction in workability has often been overcome by proposing the introduction of peculiar additives, as shown in [31,32].

In this work, the introduction of a recycled sand provided by a local company obtained from construction and demolition wastes into the mix of various kinds of mortar is discussed. In particular, three classes of mortar defined by Italian Standard [33] are investigated, varying the type of binder (hydraulic lime or cement). To the best of the authors’ knowledge, this aspect deserves further investigation in order to highlight the effect of the choice of the binder and provide new results that are able to strengthen the tendency of the introduction of recycled sand in the mixture; as a consequence, the aim is to reduce the great variability of results that are available in the literature. Each mortar, characterized by different prescribed mechanical strength parameters, is investigated increasing the percentage of recycled aggregates. In particular, in the following experimental analyses, the recycled sand is introduced in the mixes up to a total replacement of the virgin aggregates. In this framework, the water/binder ratio is kept constant. The results are presented in terms of flexural and compressive strengths. A discussion on the consistency of the fresh mixture is also included in the manuscript.

## 2. Definition and Characterization of Materials

The production of the recycled aggregates establishes that the construction wastes are collected from the demolition of buildings and infrastructures. Once the material arrives at the production site, separation of the mixed rubble and rubble consisting only of concrete waste is first performed. This classification is due to a possibility granted by the Italian Standard to manufacture new concrete structural elements embedding recycled aggregates up to a percentage equal to 30% only if more than 90% of waste is made of old concrete [33]. It should be observed that no indications concerning the production of mortar with recycled constituents are specified. Therefore, the present study is focused on the gradual replacement of the natural sand with recycled sand, which is obtained from the crushing and sieving of mixed construction wastes, which represent a significant amount of debris stored in the landfill and cause a great environmental impact.

**Figure 1:**
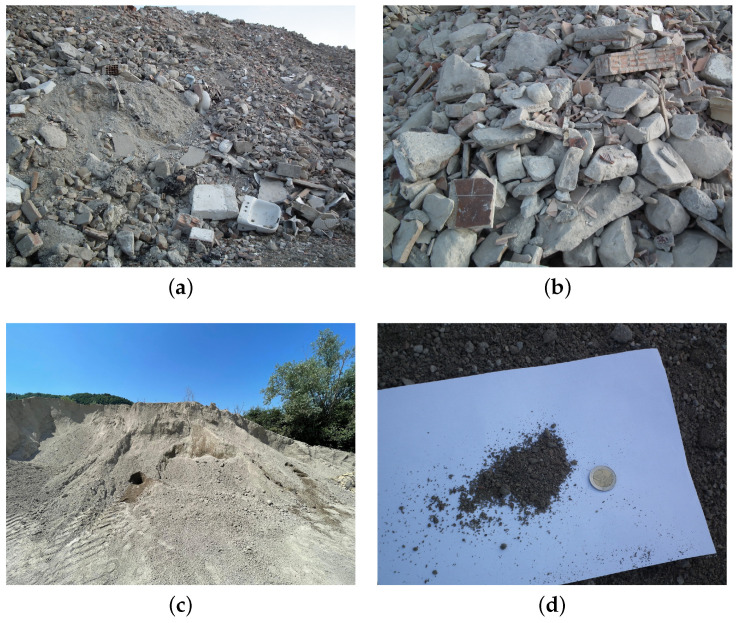
Construction and demolition wastes: (**a**,**b**) accumulation of construction wastes before the mechanical treatment; (**c**) pile of RS after the crushing and sieving processes; (**d**) particular of a RS specimen and its relative size.

In order to provide a characterization of the mechanical properties of mortars incorporating both recycled and natural aggregates, two types of sand are employed in the following tests, which are virgin or natural sand (labeled by “NS”) and recycled sand (labeled by “RS”). It is well known that NS is derived from the crushing of natural stones, resulting in a finer material with a grain size ranging from 0 to 6 mm. On the other hand, RS is obtained through the mechanical processing, consisting of crushing and sieving, of construction wastes. It is mainly formed from concrete and clay bricks, but it could also include small percentages of other residual materials, such as wood, ceramics, and plastic, as shown in Figure 1. The recycled sand used in this work is provided by A.S.A. Autotrasporti s.a. (Republic of San Marino).

In this framework, it should be specified that the local company that provided the RS has been authorized to accept the following classes of wastes: (a) cement, bricks, tiles, and ceramics; (b) wood, glass, and plastic; (c) bituminous mixtures, coal tar, and tar-based materials; (d) metals; (e) earth and rocks; (f) building materials that include gypsum.

**Figure 2:**
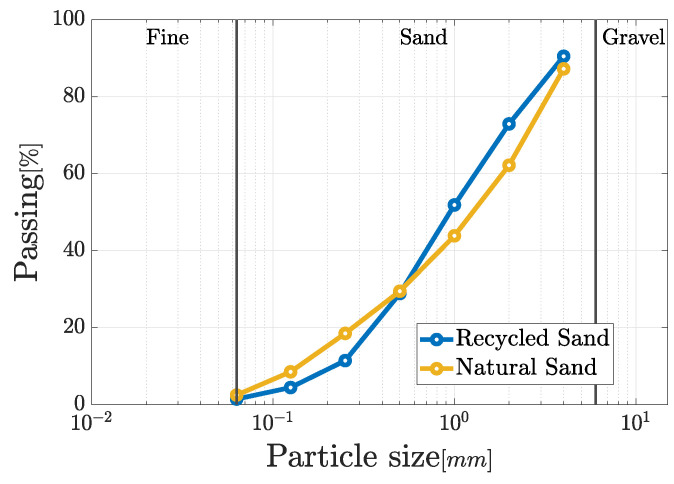
Comparison of the grain size distribution of the two types of aggregate.

The mechanical treatment used to obtain the RS provides recycled constituents characterized by a peculiar grain size distribution. In the current work, a particle range of 0–6 mm is considered to match the one that defines the NS which can be typically acquired for the preparation of mortars. In this context, the grain size distribution of the two types of sand are shown in Figure 2. The examination of the figure reveals that both types of aggregates exhibit qualitatively similar grain size distributions, proving that RS could replace NS.

A preliminary control on the acquired RS is carried out through the information given by the single-step batch leaching test (*UNI EN 12457-2:2004*), supplied by the company who provided the constituents. The analysis is performed by a certified environmental and research laboratory on a specimen of 3 kg, on 4 August 2021. Details are listed in Table 1.

The chemical and phase compositions of the RS used in the experimental tests are investigated, as well. In particular, in order to identify the minerals in the RS, an X-ray diffractometer (XRD) is used. The results provided by XRD analysis are reported in Table 2 and show that the main component is *Calcite*, followed by *Quartz* and *Albite*.

**Figure 3:**
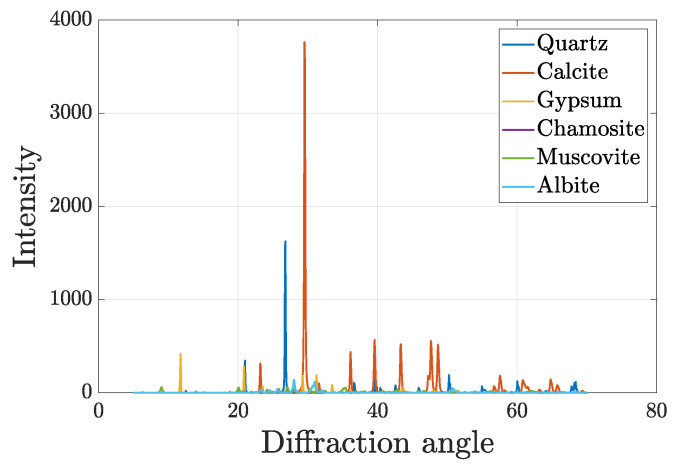
X-ray diffraction of the recycled sand.

The diffraction pattern of the sample is presented in Figure 3 for the sake of completeness.

**Figure 4:**
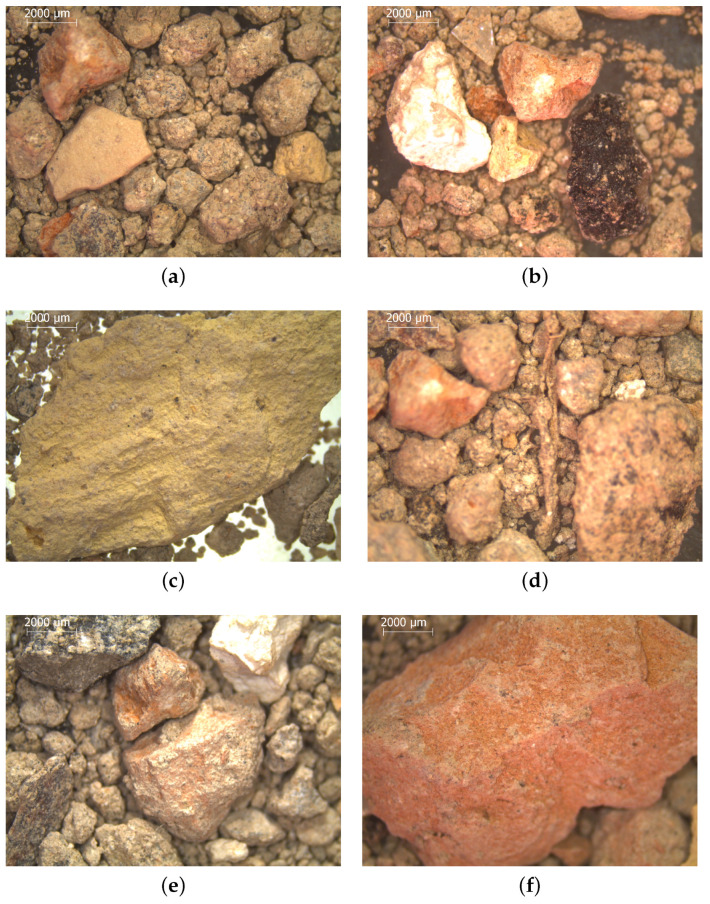
Results provided by the optical microscopy analysis: (**a**) concrete waste; (**b**) concrete and asphalt wastes; (**c**) clay or mudbrick fragment; (**d**) particular of a wooden piece surrounded by other rocky aggregates; (**e**) concrete and clay fragments; (**f**) red ceramic waste.

The microstructure of the RS has been also investigated. To this aim, an optical microscopy analysis is performed on the main part of the material, whereas an environmental scanning electron microscope (ESEM) is used on only the finer portion of the sand. The images provided by the optical microscopy are shown in Figure 4.

The observation of the optical microscopy results confirms the heterogeneity of the materials that are included in the mixed debris, which mainly consists of concrete, wood, ceramic, asphalt, and clay fragments.

**Figure 5:**
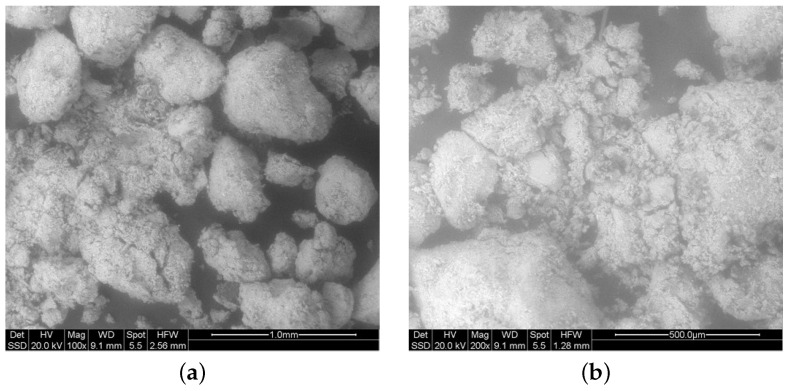
ESEM images: (**a**) 100× image (1 mm); (**b**) 200× image (500 μm).

On the other hand, the ESEM results, are presented in Figure 5. These pictures allow investigation of the micro-structure of the sand, showing that the finer part of the material is very small compared to the larger aggregates. This observation can confirm the quality control during the crushing and sieving process.

In the following, different percentages of RS are considered during the preparation of the mortar in order to investigate the effect of the gradual replacement of the NS on the mechanical properties. In particular, the relative percentages of NS and RS taken into account are listed in Table 3, together with the corresponding nomenclature for an easier identification.

These four sets are considered in the preparation of each mortar. In particular, three different classes of mortar, which are defined as M2.5 (lime mortar), M8 (lime-cement mortar), and M12 (cement mortar), are investigated. It should be noted that the number used in the previous classification defines the corresponding strength of the mortar. In other words, M2.5, M8, and M12 must be characterized by a compressive strength of 2.5 N/mm^2^, 8 N/mm^2^, and 12 N/mm^2^, respectively. According to the Italian Standard [33], mortars with prescribed compositions must satisfy peculiar volume fractions of the constituents, which are cement, hydraulic lime, sand, and water, in order to guarantee the proper compressive strength. It can be observed that two different types of binder are used: CEM II/B 32.5R and hydraulic lime. The volume fractions of the constituents for the four sets considered in this work are shown in Table 4. It should be noted that only Set 1 is included in the Italian Standard [33] since the percentage of RS is equal to 0% (the use of RS is not yet contemplated).

Overall, twelve mortar mixes have been prepared. As far as the experimental test is concerned, ten specimens are manufactured for each class of mortar for every set, following the volume fractions defined in Table 4.

**Figure 6:**
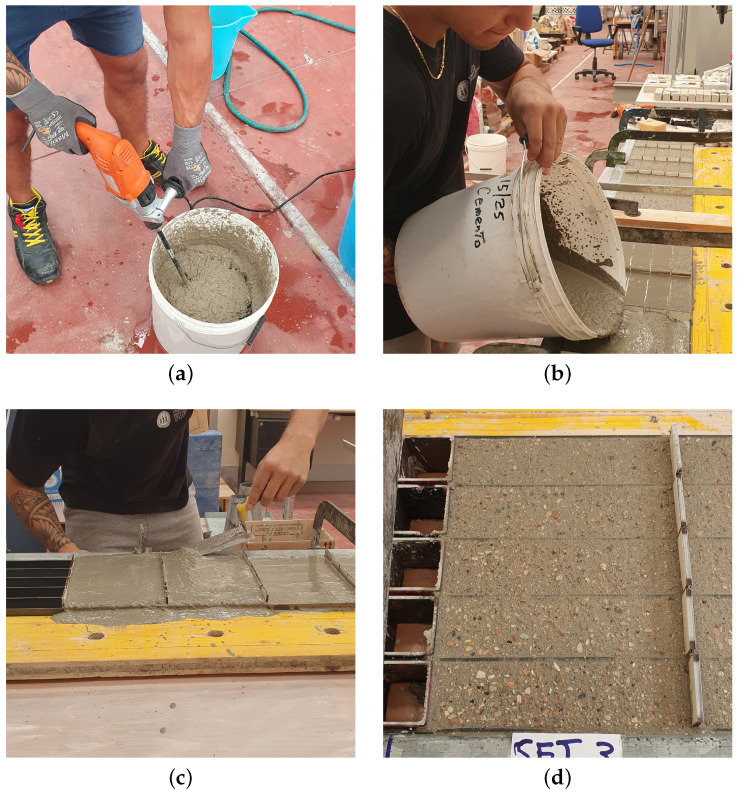
Specimen casting: (**a**) mixing phase of the process once the water is added; (**b**) pouring the fresh mortar in the mold; (**c**) specimen refinement to ensure the right geometry; (**d**) fresh mortar in the mold.

Once the mixtures are properly defined following the volume ratio in Table 4, the constituents are mixed together by an electric mixer in a dry state condition; the water is added when the mix achieves a good level of homogenization. As soon as the mixture reaches the desired state, it is poured into prismatic molds measuring 40×40×160 mm^3^. The procedure for the specimen casting is shown in Figure 6 for the sake of clarity.

It should be specified that the consistency of mixture has been also tested for each group.

**Figure 7:**
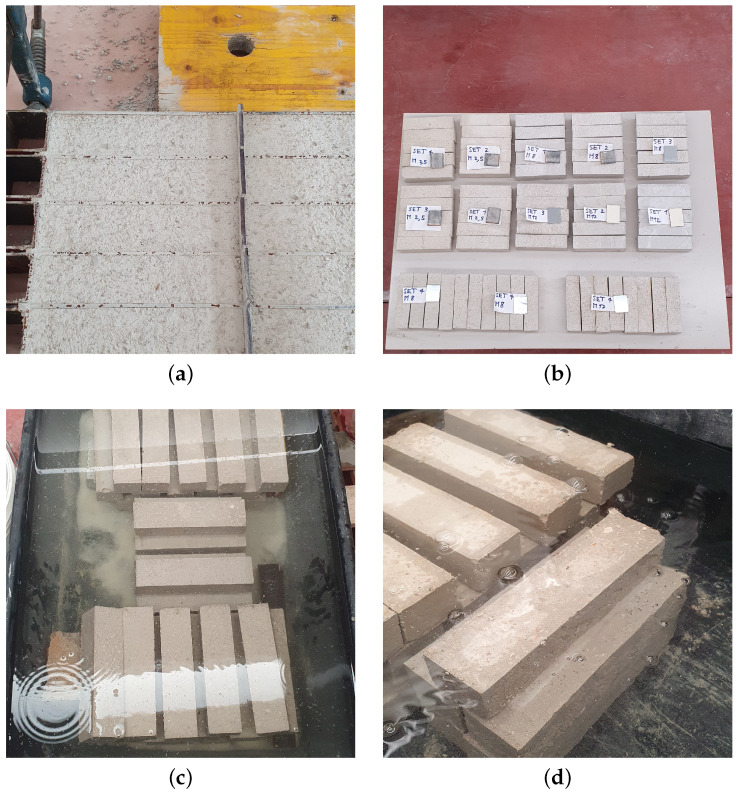
Specimen curing: (**a**) dry mortar after 2 days of curing in the mould; (**b**) specimens in air curing condition; (**c**,**d**) specimens under water curing conditions.

Following the procedures outlined in [34], the specimens are left in the molds for approximately two days. Subsequently, the specimens are divided into two groups to be cured in air and in water for 28 days to facilitate the development of the desired mechanical strength. The choice of curing half part of the specimens in air is performed to simulate the curing in typical summer environments, characterized by higher temperatures (approximately 30 ± 2 °C). On the other hand, the specimens cured in water are immersed in a water tank to guarantee the desired level of humidity (≥95%). These different curing conditions are depicted in Figure 7.

**Figure 8:**
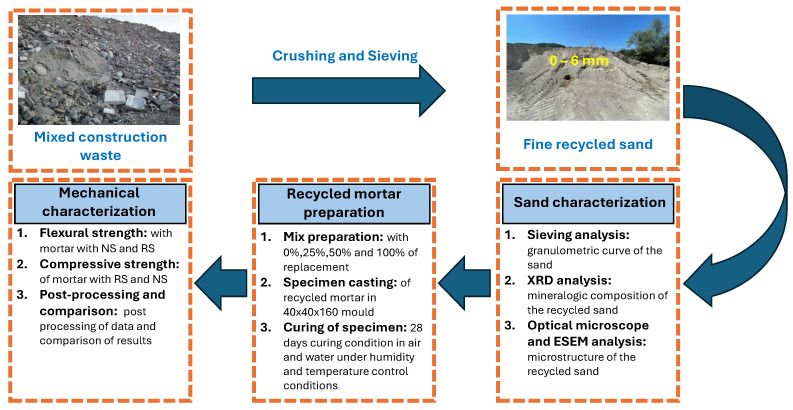
Flowchart of the experimental procedure from the construction wastes to the mechanical characterization of sustainable mortars.

Once cured, the specimens are tested to evaluate their mechanical features. The experimental workflow of this work is summarized in Figure 8 for the sake of clarity.

## 3. Methods and Test Details

The experimental analyses are carried out to measure both consistency and mechanical features of the mortars. To this aim, the slump test is performed on the fresh mortar to evaluate the consistency of the mixtures, whereas the mechanical features are evaluated on the hardened mortar specimens according to the selected procedures, which are the three-point bending test and the unconfined compression test. The main features of the procedure are briefly summarized below. The results have been carried out through the instrument MetroCom Dina960xp 600kN–type 10402060 (year 2009).

### 3.1. Slump Test

**Figure 9:**
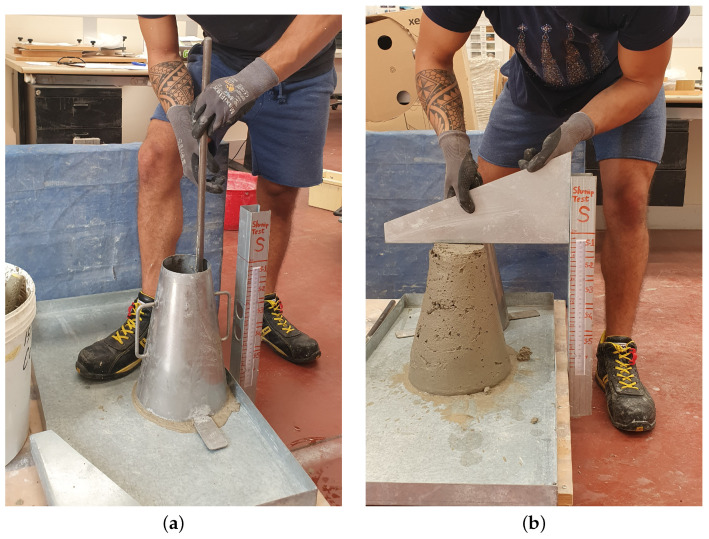
Slump test: (**a**) compacting phase of the test performed by means of a constipation bar; (**b**) measurement phase of the test.

The consistency of the mortar is determined by measuring the slump of the Abrams cone, according to the standard defined in [35]. The fresh mortar is poured in three different layers, each corresponding to one-third of the total height of a steel mold shaped like a truncated cone. Each layer of fresh mortar is compacted with 25 strokes using a tamping rod. The strokes are distributed in a spiral pattern, moving from the perimeter of the mold towards the center. After compacting the final layer, the mold is removed, and the slump is measured immediately without interruption. The higher the slump is, the lower the consistency of the sample and the higher the workability are. The compaction and the measurement phases of the test are depicted in Figure 9.

### 3.2. Three-Point Bending Test

**Figure 10:**
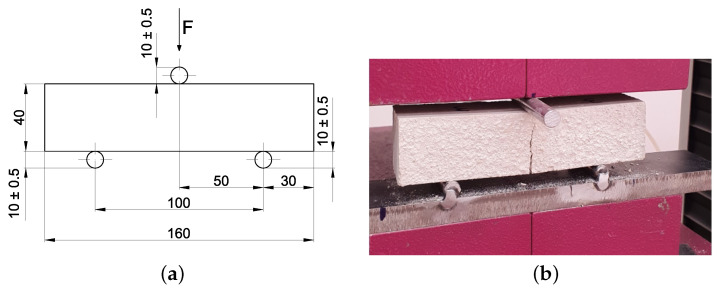
Three-point bending test: (**a**) static scheme; (**b**) failure state of the specimen on the testing machine.

A three-point bending test is first carried out to assess the flexural strength of the mortar. The well-known static scheme of the test is presented in [34], and it is shown in Figure 10 for completeness purposes together with the representation of the failure state of the specimen on the testing machine once the procedure is accomplished. The specimens are subjected to a concentrated force in the middle of the sample. This force is increased with a load rate of 0.05 KN/m according to the standard until the specimen reaches failure.

The maximum flexural strength can be computed by means of the expression below, as specified in [34]:

(1) $$f_{s}=1.5\;\cdot \;\frac{F\;\cdot \;l}{b\;\cdot \;d^{2}},$$

where $f_{S}$ is the maximum flexural strength, *l* is the distance between the support, whereas *b* and *d* are the dimensions of the specimen cross-section.

### 3.3. Unconfined Compressive Test

**Figure 11:**
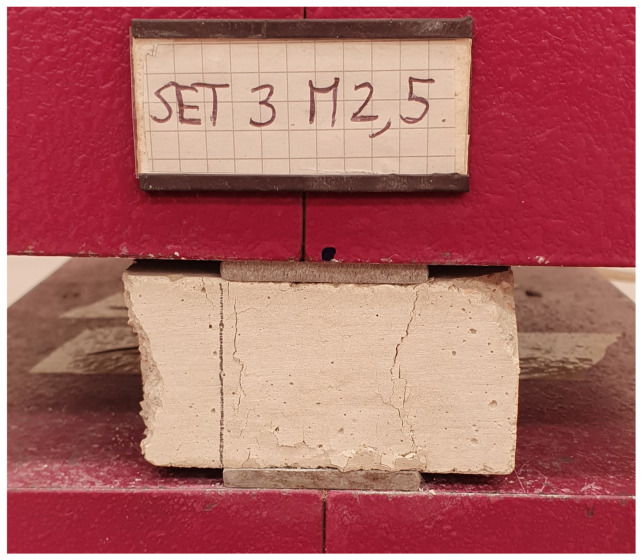
Unconfined compressive test set up and failure of the specimen under uniaxial load.

An unconfined compression test is carried out on the two pieces of the specimen once complete failure is reached in the three-point bending test since the fracture occurs in approximately the central cross-section of the samples. Therefore, the two resulting parts can be both tested. As far as the unconfined compressive test is concerned, the standard [34] is followed. The applied load *F* is orthogonal to the casting direction, with an increasing load ratio in the range 50–500 N/s, so that the failure of the specimens occurs in the time range 90–120 s. A metal thin plate with a planar size of 40×40 mm^2^ is placed on the external surfaces of the specimens to be tested to guarantee the proper load shape according to the standard [34]. For the sake of clarity, the test setup is shown in Figure 11.

The testing device collects the maximum force applied to the surface of the specimens in kN. The following expression is used for the computation of the maximum stress at which the failure occurs:

(2) $$f_{c}=\frac{F}{A},$$

where *A* is the load surface.

## 4. Results and Discussion

In this section, the main results of the experimental analyses are discussed in terms of consistency (slump test), flexural strength (three-point bending test), and compressive strength (unconfined compression test).

### 4.1. Slump Test

The results in terms of consistency are shown in Table 5 for each mix. Different categories can be defined based on slump results according to the standard defined in [35]. From the values shown in Table 5, it is possible to observe that the fluency of the mix intensifies with the increasing percentage of the recycled constituents. This aspect is clearly due to the presence of clay brick particles in the RS, which are characterized by a higher water absorption. Even if the index representative of the water absorption is not directly computed, the results of the slump tests can be taken into account to qualitatively evaluate the effect of the RS in this context. Quantitative measures of water absorption will be discussed in future works due to the practical importance of this parameter.

In general, it can be noted that Set 4 always has the highest consistency within each class; furthermore, it is observed that there is a higher setting speed compared to other mixtures, shortening the product’s workability time in comparison to mixtures containing ordinary sand or lower percentages of recycled aggregates. In addition, M8 mortars, regardless of the amount of recycled and virgin aggregates, experience the least settlement during the test. This can be attributed to the higher amount of binder in the composition compared to M2.5 and M12 mixtures.

### 4.2. Three-Point Bending Test

**Figure 12:**
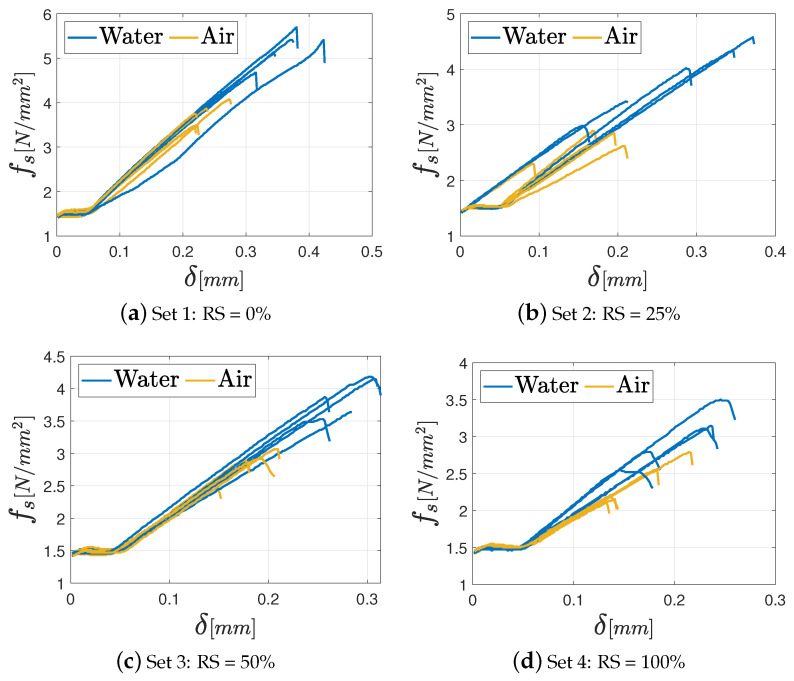
Three-point bending test results for the M8 mortar varying the percentage of RS: (**a**) Set 1: 0%; (**b**) Set 2: 25%; (**c**) Set 3: 50%; (**d**) Set 4: 100%.

**Figure 13:**
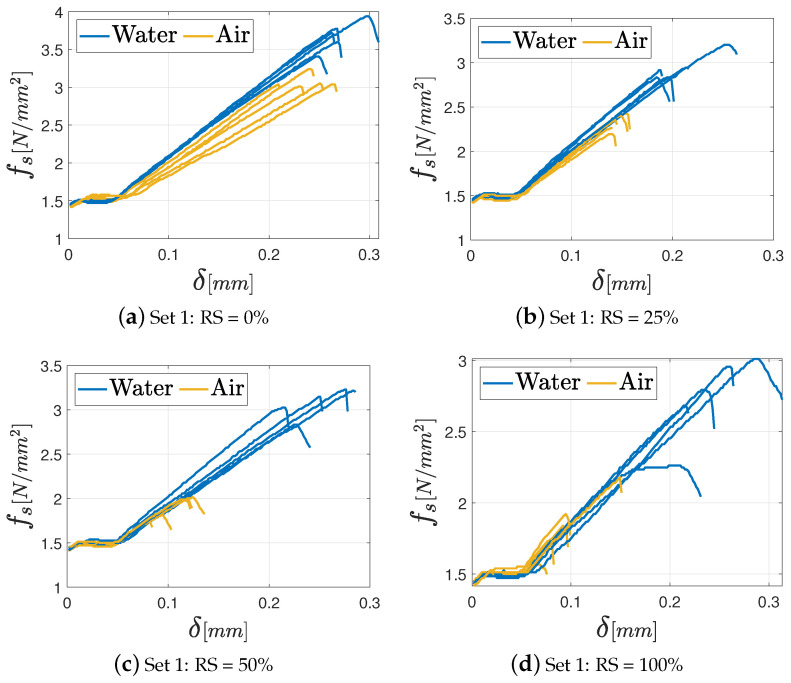
Three-point bending test results for the M12 mortar varying the percentage of RS: (**a**) Set 1: 0%; (**b**) Set 2: 25%; (**c**) Set 3: 50%; (**d**) Set 4: 100%.

As far as the three-point bending test is concerned, the results in terms of $f_{s}-\delta$ diagrams (with δ being the displacement) are presented in Figure 12 and Figure 13 for the M8 and M12 classes of mortars, respectively. The results of the M2.5 mortars are not included in this paragraph because most of the tested specimens are able to reach the minimum force required by the testing machine to generate the diagrams. In the graphs, the various curves are related to the proper curing process (air or water) to clearly identify its effect on the mechanical features.

In general, all the specimens exhibit an initial settling phase due to aggregate interlocking followed by a linear elastic phase until the maximum load is attained. In fact, once the maximum load is reached, no further strength contribution is observed in brittle materials, and the force drops quickly after that point because of the opening of a central fracture in the specimen. Regardless of the percentage of the RS in the mixture, the M8 mortar achieves the best performance under both air and water curing conditions. This outcome is closely tied to the higher amount of binder in the mix. In addition, some differences in terms of maximum strength between specimens cured in air and those cured in water can be observed. In particular, specimens cured in water yield the best results in terms of mechanical performance, especially as far as the M8 mortar is concerned. This observation is coherent with the findings presented in previous studies [36,37], which relate the superior performance under water-curing conditions to the absence of water loss during the curing process. Consequently, the formation of hydrated calcium silicate, which has the main role in the development of concrete and mortar strength, is optimized.

**Figure 14:**
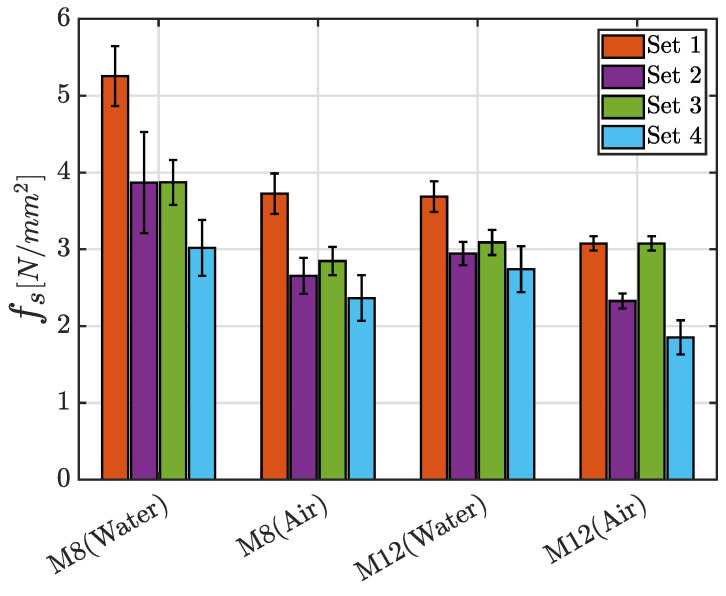
Mean value of the maximum flexural strength $f_{s}$ for each class of mortar with the corresponding standard deviation, grouped according to the adopted curing process.

A general and significant difference in terms of flexural strength can be observed by means of the comparison of the mortars with RS or NS. In fact, the use of recycled aggregates, independently from their percentages, entails a weakening of the properties if compared to the ones which characterize the Set 1 that has no RS in the mixture. This aspect is particularly evident in the M8 mortars cured in water, where a decrease about 80% is noted between Set 1 and Set 4. The other cases are characterized by a percentage difference in the range 35–55%. These considerations can be easily deduced from the bar graphs in Figure 14, where the mean values of the maximum flexural strength $f_{s}$ for each class of mortar with the corresponding standard deviation, grouped by the adopted curing process, are presented.

The inferior performance of Set 4 can be attributed to the substantial water absorption of the RS during the hydration process, leading to significant shrinkage during the curing phase. Consequently, an increased occurrence of surface cracks is noted in the specimens, resulting in a reduced strength.

### 4.3. Unconfined Compression Test

**Figure 15:**
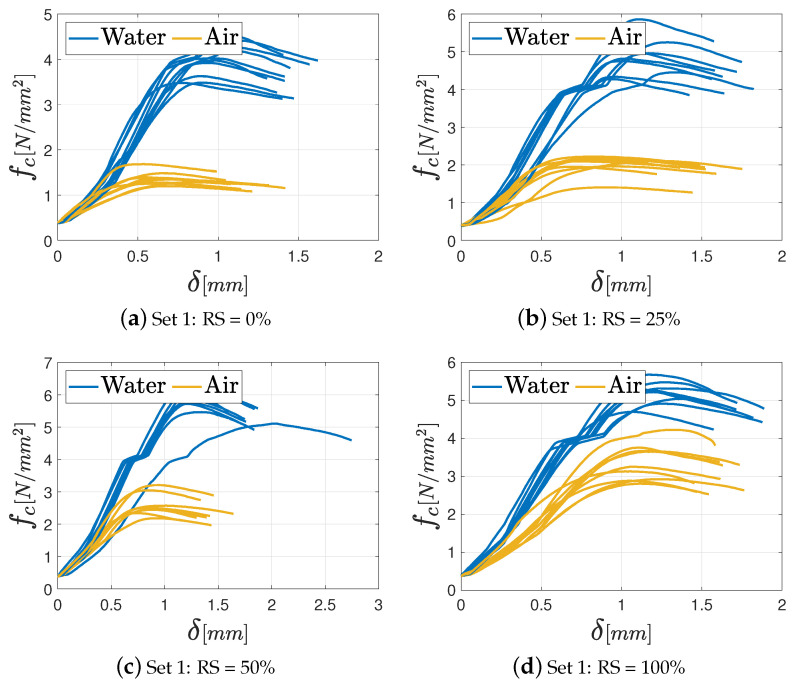
Unconfined compression test results for the M2.5 mortar varying the percentage of RS: (**a**) Set 1: 0%; (**b**) Set 2: 25%; (**c**) Set 3: 50%; (**d**) Set 4: 100%.

**Figure 16:**
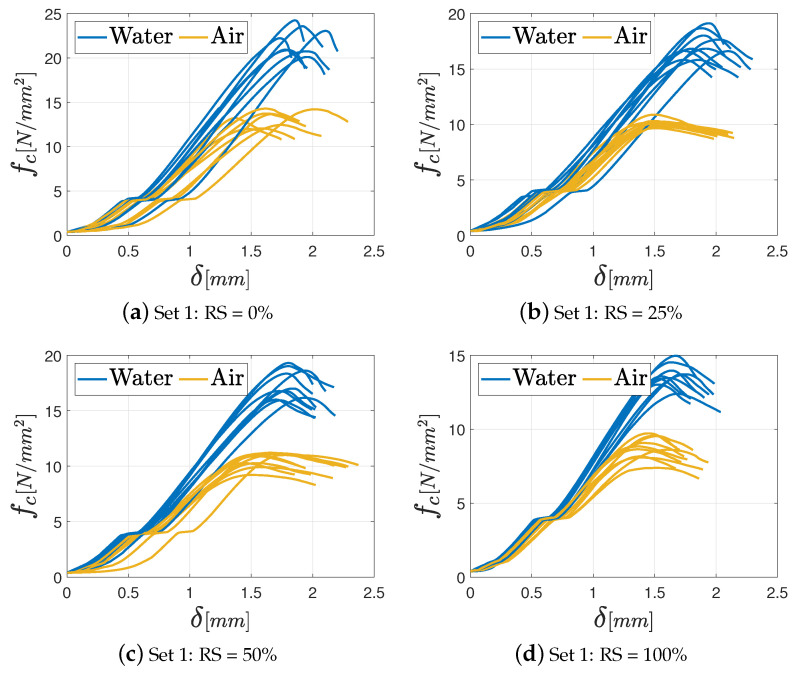
Unconfined compression test results for the M8 mortar varying the percentage of RS: (**a**) Set 1: 0%; (**b**) Set 2: 25%; (**c**) Set 3: 50%; (**d**) Set 4: 100%.

**Figure 17:**
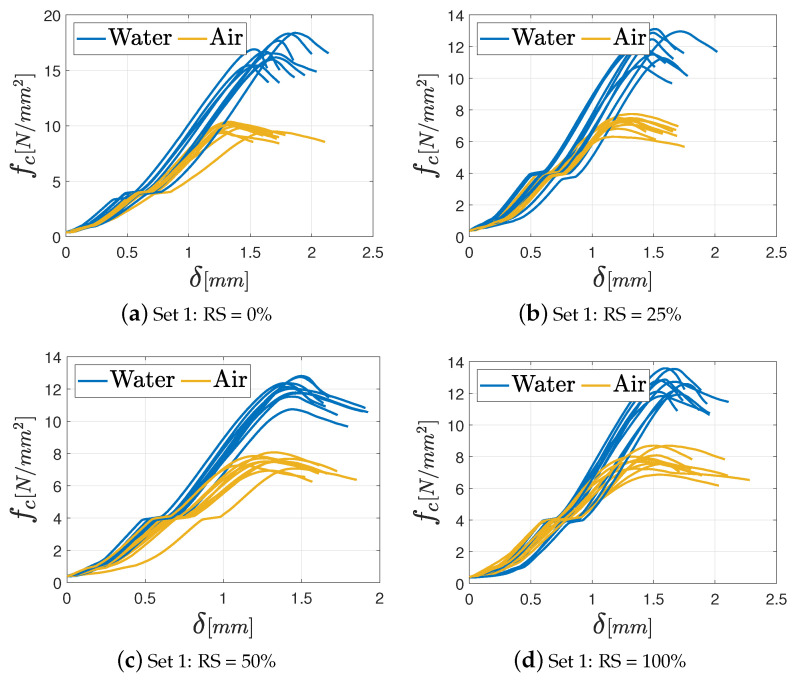
Unconfined compression test results for the M12 mortar varying the percentage of RS: (**a**) Set 1: 0%; (**b**) Set 2: 25%; (**c**) Set 3: 50%; (**d**) Set 4: 100%.

As far as the unconfined compression tests are concerned, the results in terms of $f_{c}-\delta$ diagrams (with being δ the displacement), are shown in Figure 15, Figure 16 and Figure 17, respectively, for M2.5, M8, and M12 mortars. In the graphs, the various curves are related to the proper curing process (air or water) to easily identify its effect on the mechanical features.

As in the previous tests, all the specimens exhibit a linear elastic phase until the maximum load, beyond which the force decreases gradually, marking the onset of a nonlinear elastic phase. The typical failure shape under uniaxial compression is illustrated in Figure 11.

**Figure 18:**
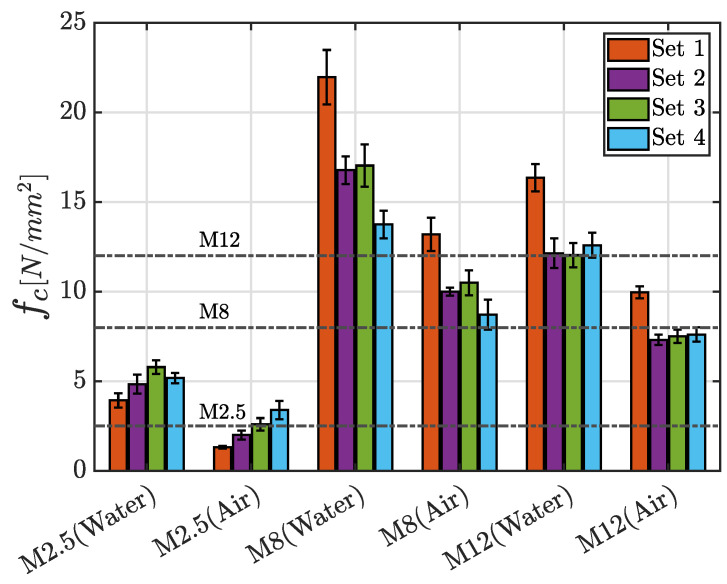
Mean value of the maximum compressive strength $f_{c}$ for each class of mortar with the corresponding standard deviation, grouped according to the adopted curing process.

Notably, the influence of curing conditions is more pronounced in the unconfined compression test, as depicted in Figure 18, where the mean value of the maximum compressive strength $f_{c}$ for each class of mortar with the corresponding standard deviation, grouped according to the adopted curing process, is presented. In general, a maximum decrease about 70% is noted between Set 1 and Set 4, as far as the M8 mortar cured in water is concerned. Except for this extreme case, a percentage reduction of 30–40% is observed for the other sets. It is important to underline that a uniform drop of the mechanical properties is observed for the M12 mortar independently of the percentage of recycled sand, for both the curing processes. A thorough examination of the bar chart corresponding to the M2.5 mortar class in Figure 18 reveals an intriguing trend: independently of the curing conditions, an increase in the recycled aggregate ratio in the mix defines an enhanced strength. This tendency, however, is not observed across the other mortar classes. These considerations can prove that the choice of binder has an influence when the RS is used in the mixture.

In the graph shown in Figure 18, the dashed black line represents the minimum strength requirement defined by regulations for a mortar of a given class to be deemed suitable for structural applications [33]. It is evident that both M2.5 and M12 mortar mixes, when cured in air, fail to meet the minimum strength threshold fixed by the standard, while the other mixes follow the specified limits. No issues in this context are observable when the specimens are cured in water.

## 5. Conclusions

The mechanical and physical performance of various sustainable mortars, identified by M2.5, M8, and M12 by the Italian Standards, in which the sand in the mixtures has been partially or totally replaced by recycled aggregates, has been investigated experimentally. The main focus has been placed on the effect of the variation in the percentage of RS, comparing the features of the mortars with those that characterize the building materials entirely made by NS when different binders are used. These aspects represent the main novelty of the work with respect to other available results. The following conclusion can be drawn:

- The slump tests have pointed out that an increasing percentage of recycled aggregates in the mixture entails a growth in terms of consistency of fresh mortars; as a consequence, the product is characterized by smaller fluidity and workability due to the greater water absorption of RS caused by the presence of clay bricks and other earthen constituents due to their porous nature.
- As far as the mechanical properties of M2.5 mortars are concerned, it has been shown that the introduction of recycled sand (RS) has improved the compressive strength. The enhancement of the mechanical features which has been observed for increased recycled aggregate content is very probably due to non-hydrated cement particles within the recycled aggregates, as specified by Neno et al. [26]. Upon contact with water, these particles could have a positive role in the chemical reactions that enhance both adhesion and strength. This effect has been particularly notable in air-cured conditions, where the best mechanical behavior has been attained at a 100% replacement ratio. In water-cured conditions, instead, optimal results have been obtained for a 50% replacement ratio.
- With regard to the mechanical properties of the other mortar classes, it can be noted that incorporating recycled sand (RS) into the mixes significantly reduces their value, especially in the initial shift from natural sand (NS) to recycled sand (RS). The strength exhibits smaller variations when increasing the RS content. This tendency is visible for each curing condition.
- The M8 mortar has achieved superior strength in both flexural and compressive tests, remarkably exceeding the minimum thresholds established by Italian Standards [33]. This performance is likely due to a more balanced binder-to-aggregate ratio, which has reduced the effects of the introduction of recycled materials.
- In general, the curing process in air has always negatively affected the mechanical process of the mortars, if compared to the corresponding curing process in water; it can be observed that the compressive strengths of some sets are slightly lower than the minimum value, if cured in air. This process, in fact, is characterized by the disadvantage of accelerating the moisture loss, especially if the environment is particularly dry, which consequently leads to a reduction in terms of strength.

Therefore, the use of recycled sand obtained from construction and demolition wastes could represent a more sustainable alternative way of conceiving structures and non-structural components. Even if the mechanical properties of the mortar could be negatively affected by the introduction of these recycled constituents—although the experimental analyses carried out in this work have proven that it is still possible to reach the minimum values of the mechanical strengths prescribed by national standards—the advantages that can be accomplished from the environmental point of view are remarkable. In the context of circular economy, the introduction of wastes into the production chain is a virtuous attitude that allows to provide new value to those materials to be disposed, providing a possible solution to the critical situation that nowadays characterizes many landfills, unable to accept additional materials.

In parallel, it should be pointed out that the proposed approach, in spite of being a preliminary study, could reduce the extreme exploitation of the soil and natural resources, as well as the consumption of energy related to the transportation of virgin constituents. Moreover, the life cycle assessment of such a sustainable building material could be performed and compared with the ones that characterize the ordinary mortar made only by natural aggregates, in order to further emphasize the environmental advantages that could be achieved. In addition, an economic study could be performed as well.

However, it should be recalled once again that the paper represents an attempt to provide a mechanical characterization of these sustainable mortars defined by different kinds of binder. As a future development, many other aspects should be investigated such as the Young modulus, density, porosity, permeability, water absorption, and durability. The lack of these parameters represents the limitation of the current work that must be surely overcome in the near future to complete their mechanical characterization of a product that could be used in some practical applications such as plasters, structural consolidation of vaults, and masonry, for instance. In addition, long-term performance under various environmental conditions should be taken into account.

Finally, it can be surely asserted that the use of recycled aggregates from construction and demolition wastes in the production of new eco-friendly mortars has remarkable potential for promoting sustainable construction practices. Nevertheless, the variability and heterogeneity of recycled aggregates presents substantial obstacles that must be faced soon.

## Figures and Tables

**Table 1:** Leaching test of the recycled aggregates made of mixed debris supplied by the company who provided the constituents. Test is performed by a certified environmental and research laboratory on a specimen of 3 kg (date of the analysis: 4 August 2021).

Parameter	U.d.M	Value	Uncertainty
Residum 105° *UNI EN 14346-1 2007 met A*	%	99.8	±4.4
Weighted raw mass *UNI EN 12457-2:2004*	g	90	
Umidity *UNI EN 14346 A 2007*	%	0.2	
Leaching volume *UNI EN 12457-2:2004*	L	0.900	
pH *UNI EN 12457-2:2004 + APAT CNR 2060 Man 29 2003*	U.ph	9.40	±0.2
Conducibility *APAT CNR IRSA 2030 Man 29 2003*	microS/cm	1100	±70
Temperature *APAT CNR IRSA 2100 Man 29 2003*	°C	21	
Nitrati (NO3) *UNI EN 12457-2:2004*	mg/L	9.7	±0.8
Fluorides *UNI EN 12457-2:2004*	mg/L	0,47	±0.07
Sulfates *UNI EN 12457-2:2004*	mg/L	690	
Chlorides *UNI EN 12457-2:2004*	mg/L	20	±2
Cyanides *UNI EN 12457-2:2004*	μg/L CN	≤10	
Barium *UNI EN 12457-2:2004 + ISO 17294-2:2016*	mg/L	0.054	±0.015
Copper *UNI EN 12457-2:2004 + ISO 17294-2:2016*	mg/L	<0.01	
Zinc *UNI EN 12457-2:2004 + ISO 17294-2:2016*	mg/L	<0.01	
Beryllium *UNI EN 12457-2:2004 + ISO 17294-2:2016*	μg/L	<1	
Cobalt *UNI EN 12457-2:2004 + ISO 17294-2:2016*	μg/L	<1	
Nickel *UNI EN 12457-2:2004 + ISO 17294-2:2016*	μg/L	<2	
Vanadium *UNI EN 12457-2:2004 + ISO 17294-2:2016*	μg/L	37	±4
Arsenic *UNI EN 12457-2:2004 + ISO 17294-2:2016*	μg/L	<1	
Cadmium *UNI EN 12457-2:2004 + ISO 17294-2:2016*	μg/L	<0.5	
Total chrome *UNI EN 12457-2:2004 + ISO 17294-2:2016*	μg/L	40	±16
Lead *UNI EN 12457-2:2004 + ISO 17294-2:2016*	μg/L	<2	
Selenium *UNI EN 12457-2:2004 + ISO 17294-2:2016*	μg/L	1.2	
Mercurio *UNI EN 12457-2:2004 + ISO 17294-2:2016*	μg/L	<0.5	±4
Asbetos *UNI EN 12457-2:2004 + ISO 17294-2:2016*	mg/L	<1	
COD (Chemical demand of oxygen) *UNI EN 12457-2:2004 + APAT CNR IRSA 5130 Man 29 2003*	mg O^2^/L	21	±9
pH *UNI EN 12457-2:2004 + APAT CNR IRSA 2060 Man 29 2003*	U.ph	9.45	±0.2

**Table 2:** Chemical composition of the RS provided by X-ray diffraction.

Mineral	Quartz	Calcite	Gypsum	Chamosite	Muscovite	Albite
Value	0.142	0.602	0.051	0.0029	0.1	0.102

**Table 3:** Definition of the four sets of mortar considered in the experimental procedure, characterized by different relative percentages of NS and RS.

Mixes	NS %	RS %
Set 1	100%	0%
Set 2	75%	25%
Set 3	50%	50%
Set 4	0%	100%

**Table 4:** Proportion of constituents by volume and correspondence with the strength class according to the Italian Standard [33]. The values related to the mixtures of various mortars are adapted to follow the percentages shown in Table 3.

Mix	Class	Cement	Hydraulic Lime	Sand	Recycled Sand	Water
Set 1	M2.5	-	1	3	-	1
	M8	1	0.5	4	-	1
	M12	1	-	3	-	1
Set 2	M2.5	-	1	2.25	0.75	1
	M8	1	0.5	3.25	0.75	1
	M12	1	-	2.25	0.75	1
Set 3	M2.5	-	1	1.5	1.5	1
	M8	1	0.5	2	2	1
	M12	1	-	1.5	1.5	1
Set 4	M2.5	-	1	-	3	1
	M8	1	0.5	-	4	1
	M12	1	-	-	3	1

**Table 5:** Slump test results performed on the fresh mortar: consistency of the mixtures according to [33].

Class	Set	Slump in mm	Category
M2.5	Set 1	>220	S5
	Set 2	>220	S5
	Set 3	210	S4
	Set 4	50	S2
M8	Set 1	190	S4
	Set 2	100	S3
	Set 3	32	S1
	Set 4	22	S1
M12	Set 1	>220	S5
	Set 2	>220	S5
	Set 3	210	S4
	Set 4	40	S1

## Data Availability

The raw data supporting the conclusions of this article will be made available by the authors on request.

## References

[1] Lucon O., Ürge-Vorsatz D., Ahmed A.Z., Akbari H., Bertoldi P., Cabeza L.F., Eyre N., Gadgil A., Harvey L.D., Jiang Y. (2014). Chapter 9—Buildings. Climate Change 2014: Mitigation of Climate Change.

[2] Ness D.A., Xing K. (2017). Toward a Resource-Efficient Built Environment: A Literature Review and Conceptual Model. J. Ind. Ecol..

[3] (2022). Concrete Is Worse for the Climate Than Flying. Why Aren’t More People Talking About It?. https://insideclimatenews.org/news/24062022/concrete-is-worse-for-the-climate-than-flying-why-arent-more-people-talking-about-it/.

[4] Koehnken L., World Wide Fund for Nature (WWF) (2018). Impacts of Sand Mining on Ecosystem Structure, Process & Biodiversity in Rivers (Executive Summary).

[5] Rentier E.S., Cammeraat L.H. (2022). The environmental impacts of river sand mining. Sci. Total Environ..

[6] Gavriletea M.D. (2017). Environmental impacts of sand exploitation. Analysis of sand market. Sustainability.

[7] Etxeberria M., Marí A.R., Vázquez E. (2007). Recycled aggregate concrete as structural material. Mater. Struct..

[8] Kou S.C., Poon C.S. (2012). Enhancing the durability properties of concrete prepared with coarse recycled aggregate. Constr. Build. Mater..

[9] Wang B., Yan L., Fu Q., Kasal B. (2021). A comprehensive review on recycled aggregate and recycled aggregate concrete. Resour. Conserv. Recycl..

[10] Curto A., Lanzoni L., Tarantino A.M., Viviani M. (2020). Shot-earth for sustainable constructions. Constr. Build. Mater..

[11] Franciosi M., Savino V., Lanzoni L., Tarantino A.M., Viviani M. (2023). Changing the approach to sustainable constructions: An adaptive mix-design calibration process for earth composite materials. Compos. Struct..

[12] Bacciocchi M., Savino V., Lanzoni L., Tarantino A.M., Viviani M. (2022). Multi-phase homogenization procedure for estimating the mechanical properties of shot-earth materials. Compos. Struct..

[13] Pucillo G.P., Carpinteri A., Ronchei C., Scorza D., Zanichelli A., Vantadori S. (2023). Experimental and numerical study on the fatigue behaviour of the shot-earth 772. Int. J. Fatigue.

[14] Vantadori S., Colpo A.B., Friedrich L.F., Iturrioz I. (2023). Numerical simulation of the shear strength of the shot-earth 772-granite interface. Constr. Build. Mater..

[15] Barbieri L., Lanzoni L., Marchetti R., Iotti S., Tarantino A.M., Lancellotti I. (2024). Shot-Earth as Sustainable Construction Material: Chemical Aspects and Physical Performance. Sustainability.

[16] Cluni F., Faralli F., Gusella V., Liberotti R. (2023). X-Rays CT and Mesoscale FEM of the Shot-Earth Material.

[17] Vantadori S., Żak A., Sadowski Ł., Ronchei C., Scorza D., Zanichelli A., Viviani M. (2022). Microstructural, chemical and physical characterisation of the Shot-Earth 772. Constr. Build. Mater..

[18] Restuccia L., Spoto C., Ferro G.A., Tulliani J.M. (2016). Recycled mortars with C&D waste. Procedia Struct. Integr..

[19] Srivastava A., Singh S. (2020). Utilization of alternative sand for preparation of sustainable mortar: A review. J. Clean. Prod..

[20] Torkittikul P., Nochaiya T., Wongkeo W., Chaipanich A. (2017). Utilization of coal bottom ash to improve thermal insulation of construction material. J. Mater. Cycles Waste Manag..

[21] Siddique R., Singh G. (2011). Utilization of waste foundry sand (WFS) in concrete manufacturing. Resour. Conserv. Recycl..

[22] Poon C.S., Shui Z.H., Lam L. (2004). Effect of microstructure of ITZ on compressive strength of concrete prepared with recycled aggregates. Constr. Build. Mater..

[23] Silva R.V., De Brito J., Dhir R.K. (2014). Properties and composition of recycled aggregates from construction and demolition waste suitable for concrete production. Constr. Build. Mater..

[24] Ledesma E.F., Jiménez J.R., Ayuso J., Fernández J.M., De Brito J. (2015). Maximum feasible use of recycled sand from construction and demolition waste for eco-mortar production—Part-I: Ceramic masonry waste. J. Clean. Prod..

[25] Ma Z., Shen J., Wang C., Wu H. (2022). Characterization of sustainable mortar containing high-quality recycled manufactured sand crushed from recycled coarse aggregate. Cem. Concr. Compos..

[26] Neno C., Brito J.d., Veiga R. (2014). Using fine recycled concrete aggregate for mortar production. Mater. Res..

[27] Dapena E., Alaejos P., Lobet A., Pérez D. (2011). Effect of recycled sand content on characteristics of mortars and concretes. J. Mater. Civ. Eng..

[28] Braga M., De Brito J., Veiga R. (2012). Incorporation of fine concrete aggregates in mortars. Constr. Build. Mater..

[29] Nasr M.S., Shubbar A.A., Abed Z.A.A.R., Ibrahim M.S. (2020). Properties of eco-friendly cement mortar contained recycled materials from different sources. J. Build. Eng..

[30] Le T., Rémond S., Le Saout G., Garcia-Diaz E. (2016). Fresh behavior of mortar based on recycled sand–Influence of moisture condition. Constr. Build. Mater..

[31] Ferro G.A., Spoto C., Tulliani J., Restuccia L. (2015). Mortar made of recycled sand from C&D. Procedia Eng..

[32] Ghorbel E., Wardeh G., Gomart H., Matar P. (2020). Formulation parameters effects on the performances of concrete equivalent mortars incorporating different ratios of recycled sand. J. Build. Phys..

[33] Ministero delle Infrastrutture e dei Trasporti (2018). Norme Tecniche per le Costruzioni (NTC 2018).

[34] (2001). Methods of Test for Mortar for Masonry—Part 11: Determiantion of Flexural and Compressive Strength of Hardened Mortar.

[35] (2001). Testing Fresh Concrete—Part 2: SLUMP Test.

[36] Cremonez C., da Fonseca J.M.M., Cury A.C.S., Ferreira E.O., Mazer W. (2022). Analysis of the influence of the type of curing on the axial compressive strength of concrete. Mater. Today Proc..

[37] Raheem A.A., Soyingbe A.A., Emenike A.J. (2013). Effect of curing methods on density and compressive strength of concrete. Int. J. Appl. Sci. Technol..

